# Supplementary material for: Coral species composition drives key ecosystem function on coral reefs
Source: Proc Biol Sci. 2020 Feb 19;287(1921):20192214. doi: 10.1098/rspb.2019.2214 (PMC7062023; doi:10.1098/rspb.2019.2214)
Supplement: Supplementary Information [file rspb20192214supp1.pdf]

Title:

Coral species composition drives key ecosystem function on coral reefs

Authors:

Laura E. Richardson [1, 2] * l.richardson@bangor.ac.uk

Nicholas A.J. Graham [1, 3]

Andrew S. Hoey [1]

[1] ARC Centre of Excellence for Coral Reef Studies, James Cook University, Townsville, QLD 4811, Australia.

[2] School of Ocean Sciences, Bangor University, Menai Bridge, LL59 5AB, United Kingdom.

[3] Lancaster Environment Centre, Lancaster University, Lancaster, LA1 4YQ, United Kingdom.

* Corresponding author

Article DOI: 10.1098/rspb.2019.2214

Supplementary Information

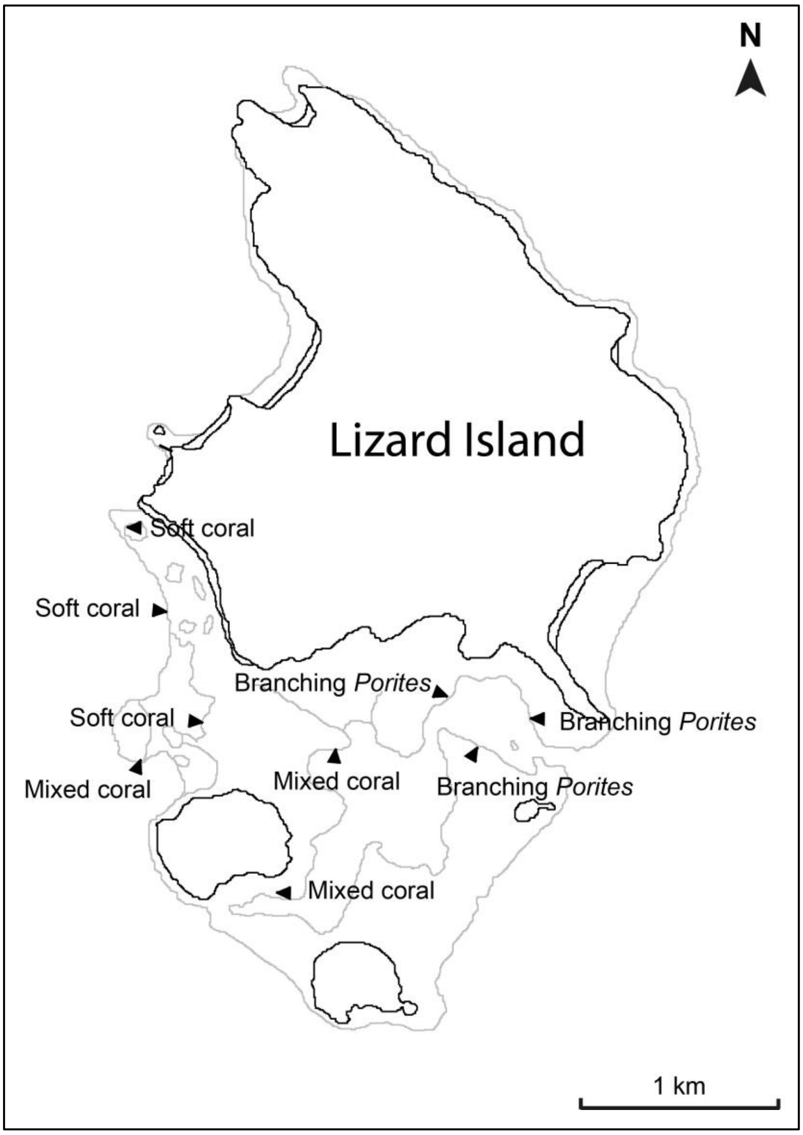

Fig. S1 Map of the location of the study sites representing branching *Porites*, mixed coral, and soft coral habitats at Lizard Island, in the northern Great Barrier Reef, Australia (14°41′S, 145°27′E).

Table S1 Surveyed species mean observed body-sizes, nominal diet groups (Green and Bellwood 2009, Wilson and others 2008), and length-weight (L-W) references used to calculate biomass of surveyed fish species.

Species	Body-size (TL; cm)	Diet group	L-W reference
Acanthurus blochii	9-12	Cropper/detritivore	Letourneur and others 1998
Acanthurus dussumieri	9-12	Cropper/detritivore	Letourneur and others 1998
Acanthurus grammoptilus	5-8	Cropper/detritivore	Froese and others 2013
Acanthurus lineatus	17-20	Omnivorous herbivore	Froese and Pauly 2014
Acanthurus nigricauda	13-16	Cropper/detritivore	Froese and Pauly 2014
Acanthurus nigrofuscus	13-16	Cropper/detritivore	Froese and Pauly 2014
Acanthurus olivaceus	9-12	Cropper/detritivore	Froese and Pauly 2014
Canthigaster amboinensis	9-12	Omnivorous herbivore	Froese and others 2013
Canthigaster solandri	≤ 4	Omnivorous herbivore	Froese and Pauly 2014
Cetoscarus ocellatus	41-44	Excavator	Froese and Pauly 2014
Chlorurus bleekeri	29-32	Excavator	Froese and Pauly 2014
Chlorurus microrhinos	9-12	Excavator	Froese and Pauly 2014
Chlorurus spilurus	9-12	Excavator	Froese and Pauly 2014
Ctenochaetus binotatus	5-8	Cropper/detritivore	Letourneur and others 1998
Ctenochaetus striatus	5-8	Cropper/detritivore	Froese and Pauly 2014
Dischistodus melanotus	≤ 4	Farmer	Froese and Pauly 2014
Dischistodus perspicillatus	≤ 4	Farmer	Froese and others 2013
Dischistodus prosopotaenia	≤ 4	Farmer	Froese and others 2013
Dischistodus pseudochrysopoecilus	≤ 4	Farmer	Froese and others 2013
Hipposcarus longiceps	33-36	Scraper	Froese and Pauly 2014
Kyphosus vaigiensis	9-12	Browser	Froese and Pauly 2014
Naso brevirostris	9-12	Cropper/detritivore	Letourneur and others 1998
Naso lituratus	37-40	Browser	Froese and Pauly 2014
Naso unicornis	25-28	Browser	Froese and Pauly 2014
Neoglyphidodon nigroris	≤ 4	Omnivorous herbivore	Froese and others 2013
Plectroglyphidodon lacrymatus	≤ 4	Farmer	Froese and others 2013
Pomacanthus sexstriatus	21-24	Omnivorous herbivore	Froese and Pauly 2014
Pomacentrus adelus	≤ 4	Farmer	Froese and others 2013
Pomacentrus brachialis	≤ 4	Farmer	Froese and Pauly 2014
Pomacentrus chrysurus	≤ 4	Farmer	Froese and others 2013
Pomacentrus grammorhynchus	≤ 4	Farmer	Froese and others 2013
Pomacentrus reidi	≤ 4	Omnivorous herbivore	Froese and others 2013
Pomacentrus simsiang	≤ 4	Omnivorous herbivore	Froese and others 2013
Pomacentrus wardi	≤ 4	Farmer	Froese and others 2013
Scarus altipinnis	5-8	Scraper	Froese and Pauly 2014
Scarus dimidiatus	21-24	Scraper	Froese and others 2013

Scarus flavipectoralis	13-16	Scraper	Froese and others 2013
Scarus frenatus	29-32	Scraper	Froese and Pauly 2014
Scarus ghobban	33-36	Scraper	Froese and Pauly 2014
Scarus globiceps	21-24	Scraper	Froese and Pauly 2014
Scarus niger	13-16	Scraper	Froese and others 2013
Scarus oviceps	17-20	Scraper	Froese and Pauly 2014
Scarus psittacus	13-16	Scraper	Froese and Pauly 2014
Scarus rivulatus	13-16	Scraper	Froese and Pauly 2014
Scarus schlegeli	29-32	Scraper	Froese and Pauly 2014
Scarus sp	≤ 4	Scraper	Kulbicki and others 2005
Scarus spinus	25-28	Scraper	Froese and others 2013
Siganus corallinus	5-8	Cropper/detritivore	Hoey and others 2013
Siganus doliatus	5-8	Cropper/detritivore	Hoey and others 2013
Siganus puellus	13-16	Cropper/detritivore	Froese and Pauly 2014
Siganus punctatissimus	13-16	Cropper/detritivore	Hoey and others 2013
Siganus punctatus	13-16	Cropper/detritivore	Froese and Pauly 2014
Siganus sp	≤ 4	Cropper/detritivore	Froese and others 2013
Siganus vulpinus	5-8	Cropper/detritivore	Froese and Pauly 2014
Stegastes apicalis	≤ 4	Farmer	Froese and others 2013
Stegastes nigricans	≤ 4	Farmer	Froese and Pauly 2014
Zanclus cornutus	5-8	Omnivorous herbivore	Froese and others 2013
Zebrasoma scopas	5-8	Cropper/detritivore	Froese and Pauly 2014
Zebrasoma velifer	≤ 4	Cropper/detritivore	Letourneur and others 1998

Table S2 Mean ± SE benthic cover of surveyed habitats in September 2015 before the April 2016 study (from Richardson and others 2017a).

Habitat	Total coral cover (%)	Dominant coral taxa	Dominant coral taxa as proportion of total coral (%)	Dominant coral taxa as proportion of total substrate (%)
Branching *Porites*	57.13 ± 2.79	Branching *Porites* (mostly *Porites cylindrica*)	78.03 ± 4.83	44.26 ± 3.39
Mixed coral	46.63 ± 3.07	-	-	-
Soft coral	59.49 ± 1.93	Soft coral (mostly *Lobophyton*, *Sarcophyton*, and *Sinularia*)	90.05 ± 2.02	53.98 ± 2.64

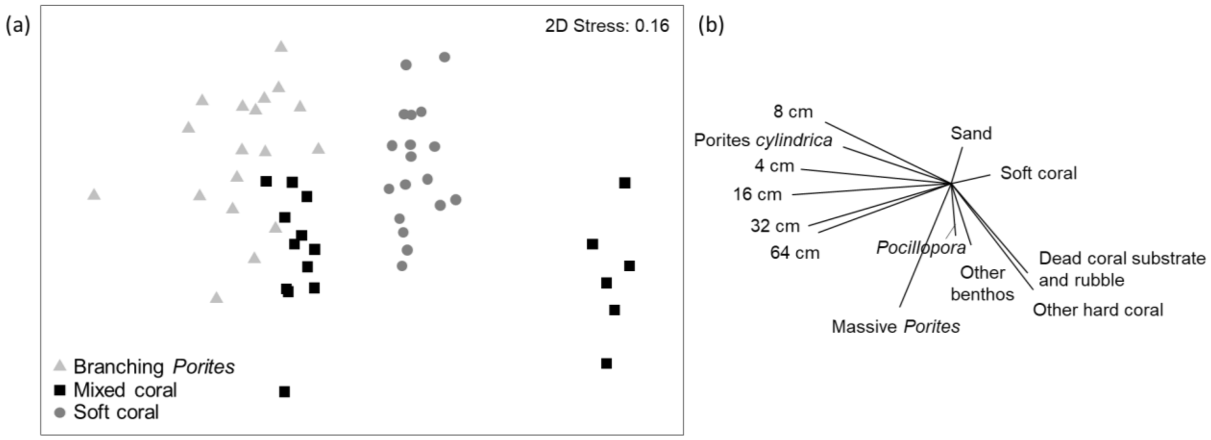

Fig. S2 Non-metric multidimensional scaling (nMDS) of spatial variation in benthic composition and structural complexity (4-64 cm scales) between the three habitat types. Structural complexity data are based on site means of 10-m transects (from Richardson and others 2017), and benthic composition from individual 30-m transects (current study). The nMDS was based on Euclidean distances of log(x+1) transformed, normalised data. (a) Data symbols represent transects within replicate sites of the three study habitat types. Vectors show the relative contribution of benthic components and scales of structural to observed variation (Pearson correlation >0.2) (b).

Table S3 Pairwise comparisons (with 95% confidence intervals: CI) among habitats, of linear mixed effects models of benthic composition and herbivorous fish assemblage structure. (Habitats: BP, branching *Porites*; Mix, mixed coral; SC, soft coral).

Response	Habitat contrast	Contrast estimate	Lower CI	Upper CI	Test stat	*df*	*P* (Tukey)
Total coral cover (hard and soft; %)	**BP > Mix**	**-16.57**	**-27.48**	**-5.67**	**-3.05**	**2, 6**	**0.01**
	BP vs SC	-10.60	-21.51	0.31	-1.95	2, 6	0.12
	Mix vs SC	5.97	-4.94	16.88	1.10	2, 6	0.51
Total bleached coral cover (hard and soft; %)	BP vs Mix	-4.31	-13.39	4.78	-0.95	2, 6	0.61
	BP vs SC	5.46	-3.62	14.54	1.21	2, 6	0.45
	Mix vs SC	9.77	0.69	18.85	2.16	2, 6	0.08
Total cover dead substrata & macroalgae (%)	**BP < Mix**	**17.22**	**3.09**	**31.36**	**2.45**	**2, 6**	**0.04**
	BP vs SC	7.69	-6.45	21.82	1.09	2, 6	0.52
	Mix vs SC	-9.54	-23.67	4.60	-1.36	2, 6	0.36
Total macroalgae cover (%)	BP vs Mix	-0.56	-1.89	0.78	-0.83	2, 6	0.64
	BP vs SC	-0.97	-2.07	0.13	-1.77	2, 6	0.17
	Mix vs SC	-0.42	-1.31	0.48	-0.94	2, 6	0.61
Herbivore species richness	BP vs Mix	1.23	1.00	1.52	1.98	2, 6	0.12
	BP vs SC	0.88	0.70	1.10	-1.16	2, 6	0.48
	**Mix > SC**	**0.71**	**0.58**	**0.89**	**-3.12**	**2, 6**	**0.01**
Total herbivore abundance	**BP < Mix**	**1.68**	**1.12**	**2.53**	**2.59**	**2, 6**	**0.03**
	BP vs SC	0.99	0.66	1.48	-0.06	2, 6	1.00
	**Mix > SC**	**0.59**	**0.39**	**0.88**	**-2.64**	**2, 6**	**0.02**
Total herbivore biomass (log-transformed; kg ha^{-1})	BP vs Mix	0.74	-0.13	1.62	1.70	2, 6	0.20
	BP vs SC	-0.35	-1.23	0.53	-0.81	2, 6	0.70
	**Mix > SC**	**-1.09**	**-1.97**	**-0.22**	**-2.51**	**2, 6**	**0.03**

Table S4 Mean ± SE UVC fish assemblage structure among habitats.

Habitat	Total herbivore biomass (kg ha^{-1})	Total herbivore abundance	Herbivore species richness	Group biomass (kg ha^{-1})	
				Browser	Grazer
Branching *Porites*	405.01 ± 105.89	129.72 ± 9.42	11.89 ± 0.69	40.85 ± 23.25	329.86 ± 103.16
Mixed coral	631.92 ± 106.56	229.10 ± 24.88	14.61 ± 0.89	24.31 ± 11.72	529.16 ± 103.69
Soft coral	206.09 ± 28.11	130.28 ± 15.13	10.44 ± 0.78	5.96 ± 3.47	163.29 ± 26.40

Table S5 Pairwise comparisons (with 95% confidence intervals: CI) of the biomass of macroalgal browsers and grazers (including croppers/detritivores, scrapers, excavators, and omnivorous herbivores) surveyed with UVC among habitats. (Habitats: BP, branching *Porites*; Mix, mixed coral; SC, soft coral).

Biomass (kg ha^{-1})	Habitat contrast	Contrast estimate	Lower CI	Upper CI	Test stat	*P* (Tukey)
Browsers	BP vs Mix	0.60	0.24	1.48	-1.44	0.49
	**BP > SC**	**0.24**	**0.08**	**0.70**	**-2.68**	**0.02**
	Mix vs SC	0.41	0.14	1.18	-1.69	0.21
Grazers	BP vs Mix	1.60	0.87	2.96	1.55	0.27
	BP vs SC	0.50	0.27	0.91	-2.31	0.05
	**Mix > SC**	**0.31**	**0.17**	**0.57**	**-3.86**	**<0.001**

Table S6 Pairwise comparisons (with 95% confidence intervals: CI) of productivity, assay change and macroalgal assay bite rates among habitats. (Habitats: BP, branching *Porites*; Mix, mixed coral; SC, soft coral).

Response	Contrasts	Contrast estimate	Lower CI	Upper CI	Test stat	*P* (Tukey)
Productivity (turf height, mm)	BP vs Mix	-0.19	-0.86	0.47	-0.58	0.83
	BP vs SC	-0.59	-1.25	0.08	-1.78	0.18
	Mix vs SC	-0.40	-1.06	0.27	-1.20	0.45
Turf removal: assay height (mm) loss	**Caged < exposed**	**-0.79**	**0.21**	**1.38**	**2.71**	**0.01**
	**BP > SC**	**2.11**	**0.73**	**3.50**	**3.04**	**0.003**
Laurencia removal: Assay wet weight (g) loss	BP: caged vs exposed	1.67	-2.00	5.34	0.90	0.37
	**Mix: caged vs exposed**	**13.41**	**6.38**	**20.44**	**3.77**	**<0.001**
	**SC: caged vs exposed**	**9.54**	**4.22**	**14.86**	**3.54**	**0.001**
Total mass-standardised bites per h	**Mix > BP**	**105.47**	**3.32**	**3347.43**	**2.70**	**0.02**
	SC vs BP	101.90	1.89	5504.42	2.32	0.05
	SC vs Mix	0.97	0.01	93.12	-0.02	0.99

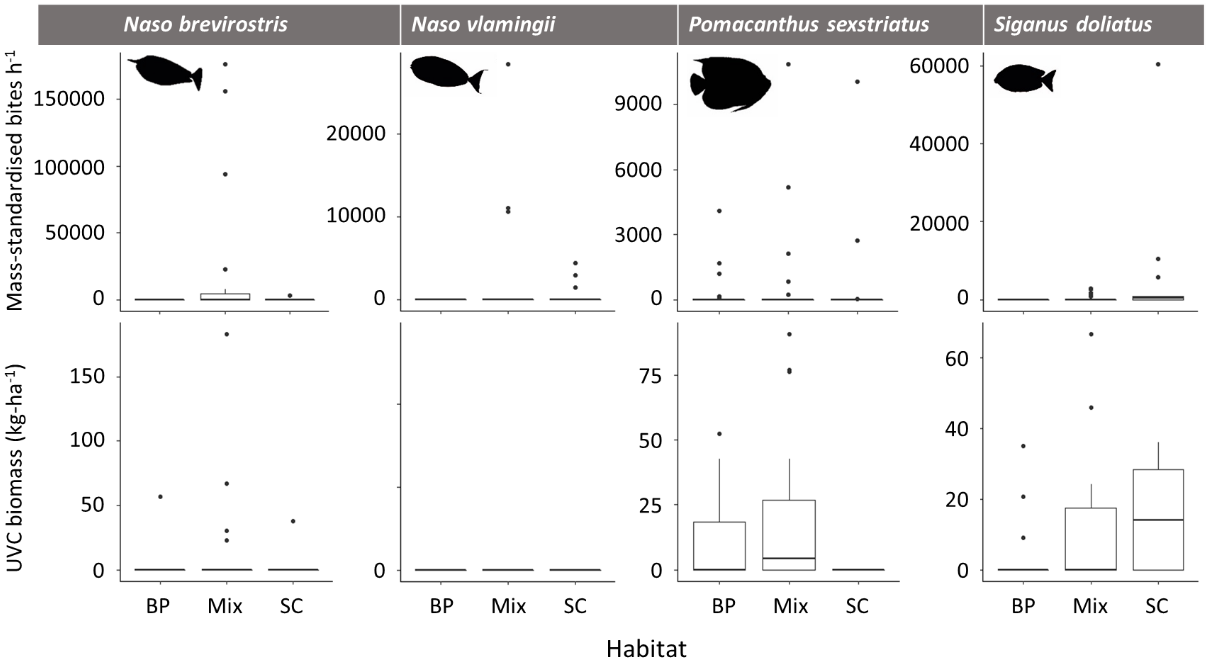

Fig. S3 Among-habitat variation in feeding rates on *Laurencia* assays (top row) by the four dominant browsing species (collectively accounting for >95% of feeding activity); and visual estimates of their biomass (bottom row). Habitats: BP, branching *Porites*; Mix, mixed coral; SC, soft coral.

Table S7 Top candidate models for predicting loss of *Laurencia* wet weight (g) (ranked within ΔAICc <2 of the most parsimonious model). The relative weight of evidence for each model is indicated by Akaike weight (wAICc), and variables present in each model are indicated (X). The relative importance of variables is based on the sum of Akaike weights of the models in which the variable is present, and parameter estimates ± standard errors and confidence intervals are provided.

Model rank	AICc	*df*	logLik	ΔAICc	wAICc	Dead substrata & macroalgae (%)	Benthic composition (PC1)	Browsers (kg ha^{-1})
1	651.56	8	-316.78	0.00	0.53	X	X	
2	651.83	7	-318.15	0.28	0.47	X		
					Model average estimate	0.51±0.20	-0.39±0.48	-
					Confidence intervals (2.5% | 97.5%)	0.04 | 0.98	-1.76 | 0.29	-
					Relative importance	1.00	0.53	-

Supporting information references

Froese R, Pauly D. 2014. Fishbase. Available at: http://www.fishbase.org

Froese R, Thorson JT, Reyes RB. 2014. A Bayesian approach for estimating length-weight relationships in fishes. *J. Appl. Ichthyol.* **30**,78-85.

Green AL, Bellwood DR. 2009. Monitoring functional groups of herbivorous reef fishes as indicators of coral reef resilience. A practical guide for coral reef managers in the Asia Pacific Region. IUCN, Gland, Switzerland. Available online at: http://cmsdata.iucn.org/downloads/resilience_herbivorous_monitoring.pdf.

Hoey AS, Brandl SJ, Bellwood DR. 2013. Diet and cross-shelf distribution of rabbitfishes (f. Siganidae) on the northern Great Barrier Reef: implications for ecosystem function. *Coral Reefs* **32**,973-984.

Kulbicki M, Guillemot N, Amand M. 2005. A general approach to length-weight relationships for New Caledonian lagoon fishes. *Cybium* **29**,235-252.

Letourneur Y, Kulbicki M, Labrosse P. 1998. Length-weight relationship of fishes from coral reefs and lagoons of New Caledonia: an update. *Naga ICLARM Q.* **21**,39-46.

Richardson LE, Graham NAJ, Hoey AS. 2017. Cross-scale habitat structure driven by coral species composition on tropical reefs. *Sci. Rep.* **7**,7557.

Wilson SK, Fisher R, Pratchett MS, Graham NA, Dulvy NK, Turner RA, Cakacaka A, Polunin NV, Rushton SP. 2008. Exploitation and habitat degradation as agents of change within coral reef fish communities. *Glob. Chang. Biol.* **14**,2796-2809.
